# High Milk Consumption Does Not Affect Prostate Tumor Progression in Two Mouse Models of Benign and Neoplastic Lesions

**DOI:** 10.1371/journal.pone.0125423

**Published:** 2015-05-04

**Authors:** Sophie Bernichtein, Natascha Pigat, Thierry Capiod, Florence Boutillon, Virginie Verkarre, Philippe Camparo, Mélanie Viltard, Arnaud Méjean, Stéphane Oudard, Jean-Claude Souberbielle, Gérard Friedlander, Vincent Goffin

**Affiliations:** 1 Inserm, U1151, Institut Necker Enfants Malades, PRL/GH Pathophysiology Laboratory, Paris, France; 2 Inserm, U1151, Institut Necker Enfants Malades, Phosphate Homeostasis Laboratory, Paris, France; 3 Pathology Department, Hôpital Necker, Paris, France; 4 Urology Department, Hôpital Européen Georges Pompidou, Paris, France; 5 Medical Oncology Department, Hôpital Européen Georges Pompidou, Paris, France; 6 Physiology Department, Hôpital Européen Georges Pompidou, Paris, France; 7 Université Paris Descartes, Sorbonne Paris Cité, Faculté de Médecine, Paris, France; 8 Assistance Publique Hôpitaux de Paris, Paris, France; 9 Institute for European Expertise in Physiology, Paris, France; Hormel Institute, University of Minnesota, UNITED STATES

## Abstract

Epidemiological studies that have investigated whether dairy (mainly milk) diets are associated with prostate cancer risk have led to controversial conclusions. In addition, no existing study clearly evaluated the effects of dairy/milk diets on prostate tumor progression, which is clinically highly relevant in view of the millions of men presenting with prostate pathologies worldwide, including benign prostate hyperplasia (BPH) or high-grade prostatic intraepithelial neoplasia (HGPIN). We report here a unique interventional animal study to address this issue. We used two mouse models of fully penetrant genetically-induced prostate tumorigenesis that were investigated at the stages of benign hyperplasia (probasin-Prl mice, Pb-Prl) or pre-cancerous PIN lesions (KIMAP mice). Mice were fed high milk diets (skim or whole) for 15 to 27 weeks of time depending on the kinetics of prostate tumor development in each model. Prostate tumor progression was assessed by tissue histopathology examination, epithelial proliferation, stromal inflammation and fibrosis, tumor invasiveness potency and expression of various tumor markers relevant for each model (c-Fes, Gprc6a, activated Stat5 and p63). Our results show that high milk consumption (either skim or whole) did not promote progression of existing prostate tumors when assessed at early stages of tumorigenesis (hyperplasia and neoplasia). For some parameters, and depending on milk type, milk regimen could even exhibit slight protective effects towards prostate tumor progression by decreasing the expression of tumor-related markers like Ki-67 and Gprc6a. In conclusion, our study suggests that regular milk consumption should not be considered detrimental for patients presenting with early-stage prostate tumors.

## Introduction

Prostate cancer (Pca) is the second most common cancer in men, with an estimated 900,000 new cases diagnosed worldwide each year [1]. European and North American countries carry the biggest burden of Pca, accounting for ~72% of the total in 2008, thus being an increasing concern of public health. Although epidemiological studies have provided strong evidence for familial (genetic) Pca, most susceptibility loci identified so far are common, low-penetrance variants with only a modest associated risk (1.10–1.25 odds ratios) [2]. Accordingly, predominant contribution to the progression of most sporadic cancers is thought to be environmental, with nutrition having a great influence [3,4].

Association between high dairy product consumption and increased Pca risk has been investigated for decades. The latest Systematic Literature Review of the World Cancer Research Fund (WCRF) International’s Continuous Update Project listed 15 studies that addressed the effect of milk consumption on Pca incidence [5,6]. Based on the inconsistency of the results (from statistically significant positive association to non-significant inverse association), it was stated that there was limited suggestive evidence that milk and dairy products increased Pca risk [5,6]. These data underline the difficulty to accurately estimate the actual impact of milk consumption on human prostate pathogenesis [7]. Accordingly, no recommendation was provided for dairy intakes since the limited evidence for Pca conflicted with decreased risk of colorectal cancer with high milk intake [5,8].

Milk is a complex mixture of various ingredients including proteins, hormones, fatty acids, calcium, vitamins, growth factors (etc.), each of which could individually contribute to Pca progression as has been suggested for calcium [9], estrogen [10], insulin-like growth factor 1 [11,12] or fatty acids [13]. Observational studies have suggested that dietary fat might contribute to Pca etiology [14], although not all studies agree on this hypothesis [15]. Since the fat content discriminates whole (high fat) versus skim (no/low fat) milk, the milk type adds a level of complexity to understanding the impact of milk consumption on prostate pathogenesis. Accordingly, while the Multiethnic Cohort Study (n = 82,483 men) did not find any association between dairy product or total milk consumption and Pca risk, further analysis revealed that low/non-fat milk consumption was moderately associated (RR = 1.16; CI, 1.04–1.29) with higher risk of localized or low-grade Pca, while whole milk consumption had an opposite effect [16]. Similar conclusions were reported in another prospective study showing that consumption of low-fat milk, but not of whole milk, was associated with increased Pca risk (RR, 1.5; CI, 1.1–2.2; P trend = 0.02 [17] and OR, 1.73; CI, 1.16–2.39; P trend = 0.0001, [18]). More recently, a prospective study reported that high intake of skim/low-fat milk was associated with a greater risk of nonaggressive Pca whereas whole milk was consistently associated with higher incidence of fatal Pca [19]. Taken together, these results suggest that lowering, or modifying, milk fat content may impact on the risk associated with the development of Pca in men, although the situation remains unclear. Accordingly, the WCRF rated the potential Pca risk associated with total fat as 'limited evidence—no conclusion' [5].

Epidemiological studies reported so far have evaluated milk consumption mainly in terms of risk (i.e. effect on cancer occurrence) more than in terms of disease progression (i.e. effect on pre-existing tumors; see Discussion). With the increasing incidence and prevalence of Pca and of benign prostate hyperplasia (BPH) worldwide due to the aging of the population and to the advent of routine prostate-specific antigen (PSA) screening, prostate tumors are increasingly considered as chronic diseases. It is therefore of interest to better evaluate modifiable risk factors that present new opportunities for prevention, including diet factors and in particular, milk. As exemplified above, it is challenging to address dietary issues via epidemiological studies in part due to measurement error, with estimates of dairy product intake based on self-reported information obtained by dietary questionnaires, and/or to confounding dietary or lifestyle factors [20]. In this respect, interventional studies involving animal models are useful to address specific questions in a more controlled environment.

**Fig 1:**
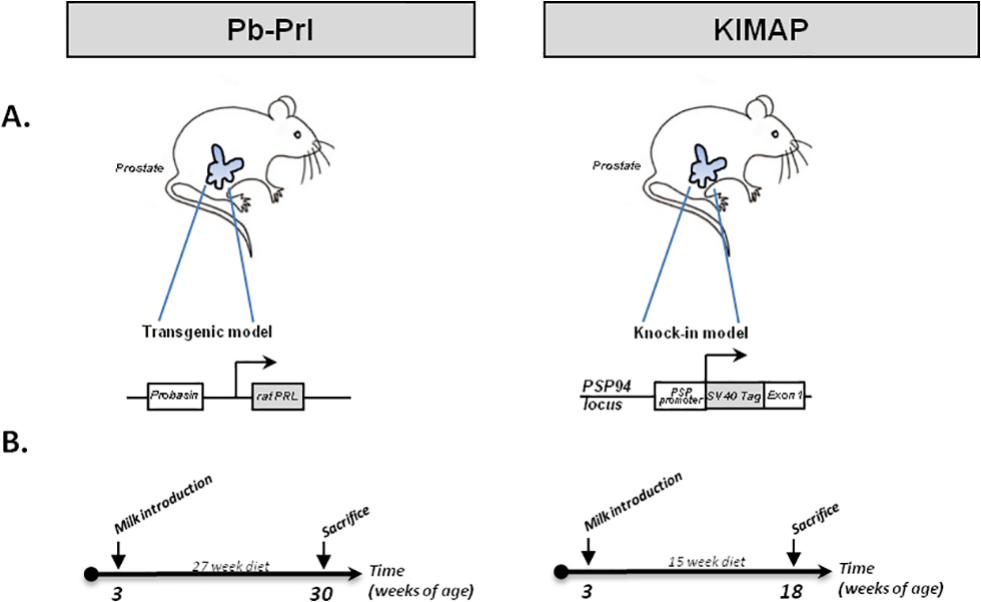
Mouse models and diet protocols. (A) Representation of the two mouse models of prostate tumorigenesis used in this study. The Pb-Prl transgenic model shown on the left involves overexpression of rat prolactin under the control of the probasin promoter. The KIMAP model shown on the right involves the knock-in of SV40 large T antigen at the PSP94 locus. Respective protocols for milk diet administration (B) are shown below each model. For both, milk was introduced at 3 weeks of age and milk diets lasted for the indicated duration. Mice were sacrificed at the end of regimens.

In this work, we aimed to determine the impact on prostate tumor progression of milk-enriched diets discriminating whole versus skim milk. To this end, we used an *in vivo* approach involving two complementary genetically-modified mouse models of early stage prostate tumorigenesis that closely mimic the human condition (Fig 1A). The first (called Pb-Prl) overexpresses the prolactin hormone specifically in the prostate [21]. This transgenic model recapitulates many features of human BPH including enlargement of all prostate lobes, marked stromal hyperplasia with moderate inflammation, ductal dilatation, focal areas of epithelial dysplasia and intra-epithelial neoplasia (PIN). The second model (called KIMAP) overexpresses the SV40 T antigen (Tag) also specifically in the prostate [22]. This model recapitulates many features of human Pca including “close-to-human” tumor progression kinetics and pathologic characteristics and highly synchronous adenocarcinoma development [23]. Both models are complementary since the Pb-Prl model is well suited to monitor the effects of the milk diets on early stages of benign tumorigenesis, whereas KIMAP mice allow investigating whether pre-neoplastic prostate lesions may evolve more rapidly to cancer-like lesions under milk regimens. Our results suggest that, in both mouse models of genetically-induced prostate tumors, high consumption of either whole or skim milk does not promote tumor progression compared to water-fed animals.

## Materials and Methods

### Animal models

The KIMAP (Knock-In Mouse Adenocarcinoma Prostate) mouse model was established by targeting the prostate-specific PSP94 locus with SV40 Tag encoding sequence, as earlier described in details [22,23]. Previous studies demonstrated that PINs were detected with high frequency at 7–11 weeks of age; by 10 weeks of age, 70% animals exhibit PIN with microinvasion; by 60 weeks of age, all mice develop solid tumor masses and by 70 weeks, metastases are detected in liver and lungs. In this study, KIMAP mice were sacrificed at 18 weeks of age, i.e. when pre-cancerous lesions are reported to evolve to cancer.

The Pb-Prl mouse model was established by additional transgenesis of the rat prolactin hormone (rPrl) encoding sequence under the control of the prostate-specific rat probasin (Pb) minimal promoter [21]. Expression of the rPrl transgene is restricted to dorsal (DP), lateral (LP), ventral (VP) and anterior (AP) prostate lobes from 4–5 weeks of age; transgene expression is undetectable in other tissues. Pb-Prl transgenic males develop significant enlargement of all prostate lobes that is evident from 10 weeks of age and increases with age [21,24]. This transgenic model recapitulates many features of human BPH including significant stromal hyperplasia, ductal dilatation, focal areas of epithelial dysplasia and low grade PINs. At >1 year of age, high grade PINs and very rare adenocarcinomas have been observed [24].

For both strains, the mice used in this study were on C57/Bl6 genetic background and hemizygous for the transgene.

### Mouse housing and sacrifice

This study was approved by the Comité d'Ethique en matière d'Expérimentation Animale Paris Descartes—CEEA 34 (authorizations # P2.VG.167/10 for KIMAP mice and 168/10 for Pb-Prl mice) and was carried out in strict accordance with the European Directive 2010/63/UE on the protection of animals used for scientific purposes. Mice were housed in polycarbonate cages in an environment-controlled room at 22°C on a 12-hour dark/light cycle and were regularly checked for signs of distress during the course of the study. Mice were sacrificed at the end of the treatment by cervical dislocation. To isolate the prostate, dissection of the urinary tract was performed and left lobes were separately dissected and snap frozen while the remaining right half of the prostate was fixed in paraformaldehyde (PFA) without being further dissected, so that tissue organization was preserved for histological analysis. We analyzed the three most commonly-studied prostate lobes (VP, LP and DP) for both models whereas AP was available for Pb-Prl mice only.

### Milk diets

Male mice were fed with manufactured animal diet (ref # 2018, Teklad Global 18% Protein Rodent Diet Harlan, USA) and water ad libitum. Milk used in this study was purchased during 2011–2012 as 750g commercial packs (Regilait, France) containing non-supplemented skim or whole milk powder (no specific reference number was available for these two products referred to as 'lait écrémé' and 'lait entier', respectively). Diet was administered every other day in a controlled manner (i.e. not *ad libitum*) in the form of crushed chow mixed with powdered milk resuspended in water (no milk powder for the control group). This way of milk administration was preferred over drinking milk to ensure equal and homogenous intake amongst animals and also to limit gastrointestinal issues by allowing mice to drink water *ad libitum*. In practice, animals received the equivalent of 5 g of chow/day/mouse and 1.4 g of milk powder/day/mouse (equivalent to 10mL reconstituted milk). Diet composition is detailed in Table 1 (quantity and caloric density in part A, milk composition in part B). Before the full study, diet acceptance was assessed in preliminary experiments using KIMAP males (three weeks-old, 10 week regimens, n = 15 animals). For each mouse model, thirty mice were randomized into 3 nutritional groups of 10 mice. Milk (or control) diets started at 3 weeks of age and lasted for fifteen weeks (KIMAP) or twenty-seven weeks (Pb-Prl model) (Fig 1B). Mice were weighed once a week during the course of the diet.

**Table 1:** Composition of diets.

**A. Dietary intake and energy density parameters**	Units	Water (Control)	Skim milk	Whole milk
**Chow contribution**	g/day/mouse	5	5	5
*Caloric density*	kcal/g	3.1	3.1	3.1
*Mean caloric intake*	kcal/day/mouse	15.5	15.5	15.5
**Milk contribution**	g/day/mouse	0	1.4	1.4
*Caloric density*	kcal/g	0	3.56	4.94
*Mean caloric intake*	kcal/day/mouse	0	4.98	6.91
**Water to reconstitute diet**	g/day/mouse	10	10	10
**Total diet**	**g/day/mouse**	**15**	**16.4**	**16.4**
*Caloric density*	**kcal/g**	**1.03**	**1.25**	**1.37**
*Mean caloric intake*	**kcal/day/mouse**	**15.5**	**20.48**	**22.41**
**B. Milk composition**	Units	Water (Control)	Skim milk	Whole milk
Energy	kcal/100g	/	356	494
Lipids (among saturated fatty acids)	g/100g	/	0.8 (0.5)	26.2 (16.5)
Glucids	g/100g	/	51.7	38.6
Proteins	g/100g	/	35.5	26
Sodium	g/100g	/	0.51	0.39

### Antibodies and oligonucleotides

References and conditions of use of the various antibodies that were used for immunohistochemical studies are listed in Table 2. Primers (Oligold quality) were from Eurogentec (Liège, Belgium) or from Integrated DNA Technologies (HPLC purification—IDT, Leuven, Belgium) as indicated. Primer sequences are listed in Table 3.

**Table 2:** List of primary antibodies used for IHC.

	Species	Ref / clone	Supplier	Dilution	Antigen retrieval conditions	Incubation
**CD45**	rat monoclonal	sc-53665 / 30-F11	Santa Cruz	1/150	Citrate 30' 95°C	O/N, 4°C
**c-Fes**	goat polyclonal	sc-7670 / C19	Santa Cruz	1/300	Citrate 30' 95°C	O/N, 4°C
**Ki-67**	rabbit monoclonal	RM9106 / SP6	Thermo Scientific	1/300	Citrate 30' 95°C	O/N, 4°C
**P-Stat5**	rabbit monoclonal	9359/C11C5	Cell Signaling	1/300	Citrate 30' 95°C	O/N, 4°C
**p63**	mouse polyclonal	sc-8431 / 4A4	Santa Cruz	1/150	Citrate 30' 95°C	O/N, 4°C
**α-sma**	mouse monoclonal	A2547 / 1A4	Sigma	1/10 000	Citrate 30' 95°C	O/N, 4°C
**SV40T**	mouse monoclonal	55–4149	BD Biosciences	1/50	Citrate 30' 95°C	O/N, 4°C

**Table 3:** List of primers used for q RT-PCR.

Eurogentec	Species	Gene	Name	5'-3' sequence
	*mouse*	***C-fes***	Mouse c-Fes—F	TTTGTAGAAAAGGGGCATCG
			Mouse c-Fes—R	GTCTCTGCCCAGGCTCATAG
	*mouse*	***Ki-67***	Mouse Ki67—F	AAAGGCGAAGTGGAGCTTCT
			Mouse Ki67—R	TTTCGCAACTTTCGTTTGTG
	*mouse*	***PSP94***	PSP94-F	TGG TGA TAG CAT CCA AAG CA
			PSP94-R	GCT TGT TAC CAT CAG CAT CC
	*mouse*	***Cyclophilin A***	Cyclo-F	CAGGTCCTGGCATCTTGTCC
			Cyclo-R	TTGCTGGTCTTGCCATTCCT
	*virus*	***SV-40 T***	SV40T- F	TGCCTGGAACGCAGTGAGTTTT
			SV40T-R	AACTCAGCCACAGGTCTGTACCAA
**IDT**	**Species**	**Gene**	**Name**	**IDT reference**
	*mouse*	***Gprc6a***	Gprc6a	Mm.PT.58.41809220

### Histology and immunohistochemistry

Tissues were fixed in 4% PFA in PBS overnight then in 50% ethanol before being processed for histological studies. Serial sections (4μm thickness; 1 section per slide) were performed as described below to ensure whole tissue screening. Briefly, three to six tissue levels of ten slides each were cut, each level being separated by 40 μm. The two first slides were kept for histology and the following for immunohistochemistry (IHC). For histology, sections were stained with classical haematoxylin-eosin (H&E). Fields were selected following systematic random sampling scheme. For IHC studies, paraffin-embedded/PFA-fixed sections (4μm) were deparaffinized in a xylene substitute (Neo-Clear) and rehydrated in graded ethanol. Endogenous peroxidase activity was blocked by incubating slides in 3% H_2_O_2_ for 10 min at room temperature, and non-specific binding of immunoglobulins was minimized by pre-incubation with 2% normal serum in PBS for 30 min at room temperature. Sections were boiled 30 min in citric acid (pH 6.0) for antigen retrieval. The avidin biotin immunoperoxidase system was used to visualize primary antigen-antibody complexes (Vectastain Elite ABC kit; Vector Laboratories, Burlingame, CA) using 3,3’-diaminobenzidine as chromogen (SK-4100; Vector). Slides were then counterstained with haematoxylin.

### Prostate histopathology

Histopathological diagnosis of all prostate sections was performed in blind by two independent pathologists (P.C., V.V.) according to specific criteria reported in earlier studies [21,24] and following the recommendations of the Mouse Models of Human Cancer Consortium Prostate Pathology Committee and the reference classification of PIN lesions in genetically-modified animals [25,26].

### Image acquisition and histology quantifications

Digital scanned images were acquired using a NanoZoomer-2.0 RT scanner (Hamamatsu, Photonics, France) coupled to NDP.view2 software analysis beta version U12388-01 (Hamamatsu, Photonics, France). All quantifications mentioned below (except for *Inflammation* where counting was performed on the entire prostate) were performed on each individual lobe of at least 6 animals from each diet group. Results are expressed as means ± S.D, corresponding to individual lobes.

#### Proliferation Index, Stat5 activation and nuclear p63 staining

The proliferation index (PI) was calculated from Ki-67 antigen staining. Briefly, PI was determined as the ratio of Ki-67 positive / total number of nuclei in the epithelium, and this was expressed as percentage. For each lobe, several fields (selected to be representative of the lobe based on H&E analysis) were counted to achieve ~9,000 to ~12,000 nuclei per mouse (~1,200–1,500 cells counted on each field). Quantification of pStat5 and p63 were performed and quantified in the same way.

#### Invasion

For quantification of invasiveness, IHC staining with anti-α-SMA antibody was performed and each discontinuous α-SMA-staining pattern surrounding glands (i.e. membrane breakage) was counted per lobe as previously described [27]. Results are represented as the number of membrane breakage per entire lobe for all diet groups.

#### Inflammation

CD45 antigen is a transmembrane glycoprotein broadly expressed among differentiated hematopoietic cells except erythrocytes and plasma cells. CD45 was originally called *leukocyte common antigen*, and is now used in routine IHC as a marker of inflammation. The degree of prostatic inflammation was evaluated using CD45 IHC. Since inflammatory cells are often grouped within clusters, the number of CD45-positive cell clusters per half prostate embedded-tissue was recorded (reported in fold expression *vs*. the vehicle group).

#### Fibrosis

For evaluation of fibrosis, picrosirius red staining was performed and areas of dense stained foci were measured in all lobes (mm^2^). The ratio of the total fibrotic areas *vs*. the total stroma area was expressed as percentage. The total stroma area was calculated by subtracting the “total lobe area” by the “total acini area” (as measured by circling each lobe and each acini within lobes for all animals).

### Quantitative RT-PCR

Total RNAs were isolated from separate prostate lobes using the NucleoSpin RNA XS (Macherey Nagel, Hoerd, France) according to manufacturer’s instructions. RNA integrity was assessed on Agilent BioAnalyzer (all RINs scored 7–10). RNA (250ng) was reverse transcribed using SuperScript II Reverse transcriptase with the SuperScript II First-Strand Synthesis System for RT-PCR kit (Invitrogen, CA, USA). For qPCR analysis, the cDNA was then subjected to real-time PCR amplification using gene specific primers and LightCycler 1536 DNA Green Master Mix (Roche Applied Science). Primers were used at a 250 nM final concentration. PPIA (Peptidyl Prolyl Isomerase A) that encoded Cyclophilin A was used as a housekeeping gene in each reaction. Real-time PCR was performed using a “LightCycler 1536 Real-Time PCR System” (Roche Applied Science, France) coupled to an Agilent Bravo Automated Liquid Handling Platform (Agilent, France). The qPCR reaction contained 0.8μL cDNA sample (1 ng) and 1.2 μL mastermix with 1X RealTime ready DNA Probes Master(Roche), 250 nM primer and 400 nM probe (Universal ProbeLi-brary, Roche). The LightCycler 1536 Instrument was used with the following program: Enzyme activation: 95C, 1 min; ampli-fication (45 cycles): 95C, 1 sec (ramp: 4.8C/s), 60C, 30 sec (ramp: 2.5C/s); cooling: 40C, 30 s (ramp: 2.5C/s). Results were generated with the LightCycler 1536 software, and were analyzed by the comparative cycle threshold method and presented as fold change in gene expression relative to internal calibrators as mentioned in Figures. Experiments were performed in duplicate and the results are expressed as means ± S.D.

### Statistics

All quantitative data are expressed as mean ± S.D and all comparisons were made with at least 6 animals per groups (unless specified) using One way ANOVA followed by Tukey’s comparison test (version 5.00, GraphPad Software, San Diego USA, www.graphpad.com). P-values between milk diet groups and control water are represented on Figures with the following symbol (*) as follows: one symbol when, p<0.05, two symbols when p<0.01 and 3 symbols when p<0.001.

## Results

### Diet tolerance and prostate hypertrophy

This study was performed to address whether milk consumption (skim or whole) may affect prostate tumor progression. To that end, two complementary genetically-modified mouse models of prostate tumorigenesis were used (Fig 1A). Consistent with the kinetics of tumor development in each model, the milk regimens described in the Methods section lasted for 15 (KIMAP) or 27 (Pb-Prl) weeks (Fig 1B).

#### Pb-Prl mice

**Fig 2:**
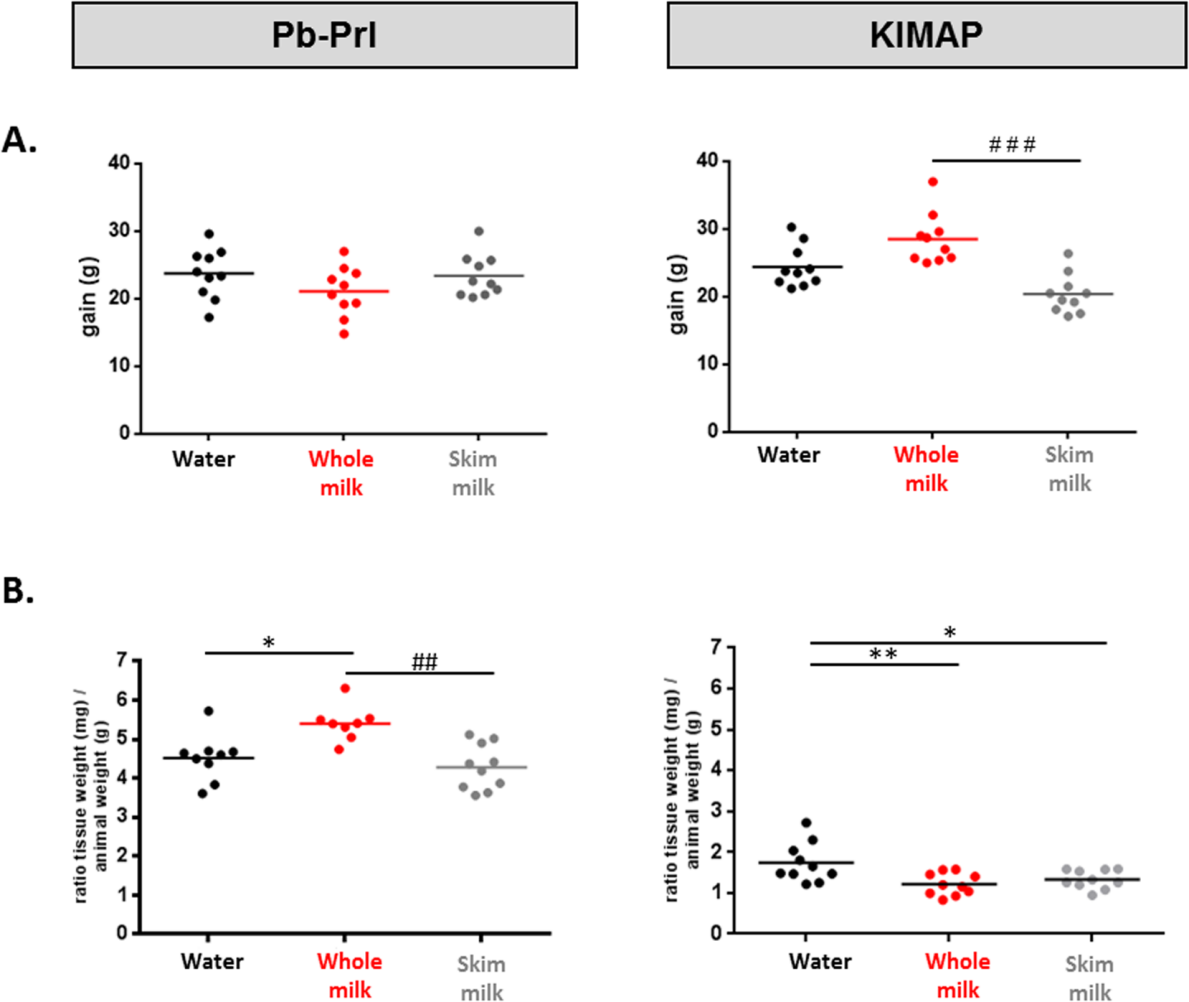
Animal weight gain and prostate weight following milk diets. Animal weight gain between 3 weeks of age (i.e. at the beginning of the different diets) and sacrifice (A) and prostate weights at sacrifice (B) are reported for Pb-Prl and KIMAP mice in all diet groups. Animal weight gain is expressed in grams (g) while prostate weights are represented as the ratio between half prostate weight and mouse weight (g). Each dot represents one animal (n = 10 mice per group); horizontal lines represent the mean of the group. P values are represented *vs*. water control group (*), or *vs*. whole milk group (#).

Mouse body weights were measured weekly between 3 weeks of age and sacrifice (30 weeks of age) (S1 Fig) As shown in Fig 2A, all mice gained weight within the expected range with no differences between the various diet groups. At the age of sacrifice (30 weeks), Pb-Prl mice were reported to harbor a hyperplastic prostate with massive enlargement of all lobes [21,24]. This was consistent with our macroscopic observations at dissection, which revealed hypertrophied prostate phenotype in all diet groups, with no obvious size differences among groups. To confirm this observation, prostate weights (measured as one half prostates, i.e. 3 non-separated lobes) were monitored immediately after dissection (see Methods). When values were normalized to animal weight, the prostate weight gain was slightly but significantly higher (~1.2 fold) in whole milk compared to the two other groups (Fig 2B).

#### KIMAP mice

As noticed for Pb-Prl mice, KIMAP mice also gained weight within the expected range with no differences between the milk diet groups and the control group (Fig 2A and S1 Fig). Of note, the net weight gain was nevertheless significantly higher in the whole milk diet group compared to the skim milk diet group (Fig 2A). At the age of sacrifice (18 weeks), KIMAP mice were reported to harbor localized prostate neoplasia with no documented impact on prostate weight [22]. This was consistent with our macroscopic observations which failed to reveal prostate hypertrophy in any group at sacrifice. Although prostate weight gain (normalized to animal weight) was slightly lower in both milk groups compared to water-fed animals (Fig 2B), it is fair to stress that such small differences must be interpreted with much caution in a model devoid of prostate hypertrophy, as dissection issues may impact on weight measurements more than any inter-animal variations. Accordingly, our results should be viewed only as evidence that milk diets did not induce prostate growth.

### High milk consumption does not aggravate prostate tumor histopathology

We systematically characterized all prostate lobes individually for both models. Of note, DP is usually considered as the most relevant lobe to the human prostate, although caution is required in making mouse-to-human extrapolation for this organ. For clarity of the results, DP was used to illustrate histopathological features in the main text Figures, but quantifications refer to the whole prostate (all lobes combined). Quantitative and illustrative data collected for each lobe individually are found in Supplementary Information.

#### Pb-Prl mice

**Fig 3:**
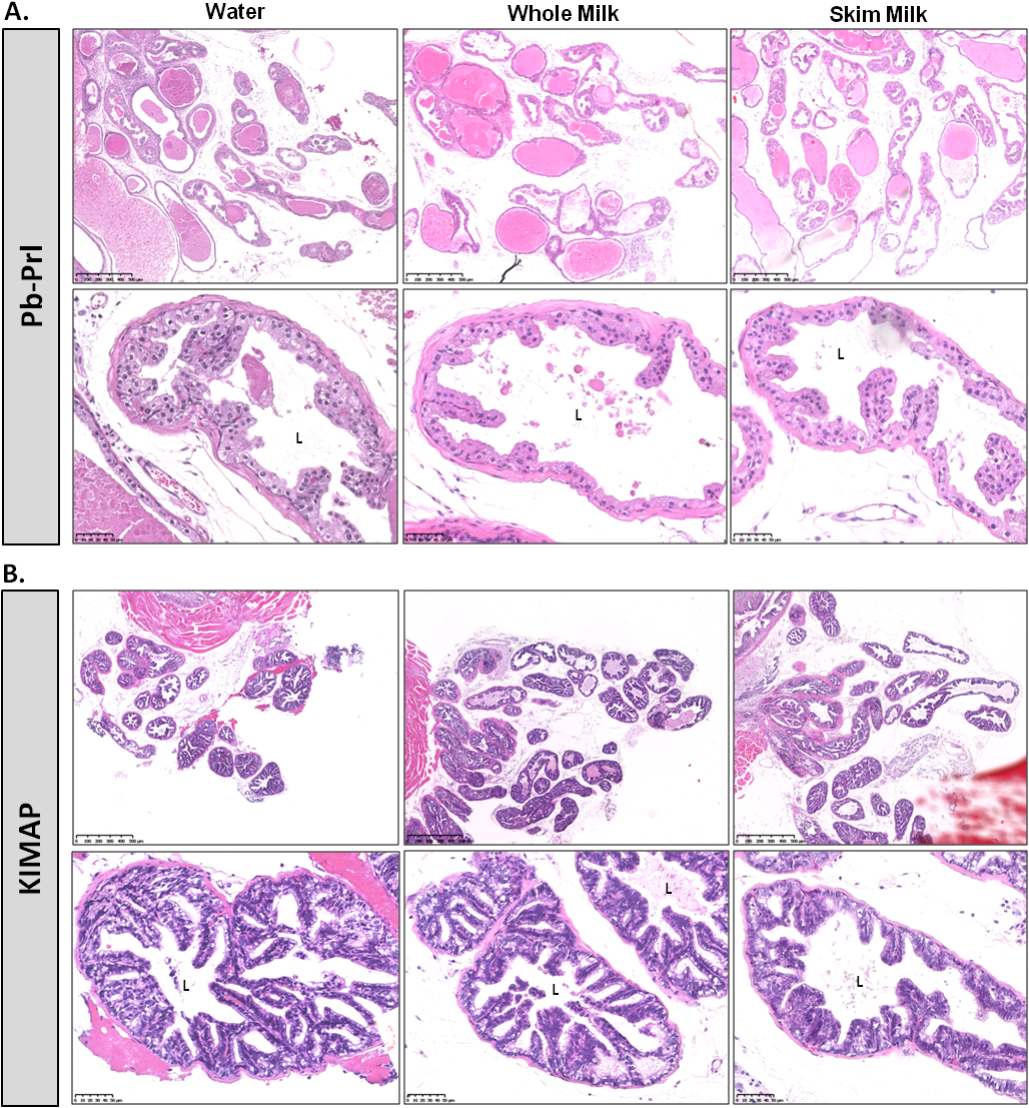
Effect of milk diets on prostate tumor histopathology. Histological analysis of dorsal prostate in Pb-Prl (A) and KIMAP mice (B) after respectively 27- or 15-week milk diets, as compared to control (water) group. For each model, images are representative of the group (n = 10 mice per group). Sections were stained with haematoxylin/eosin (HE) and for both models, upper pictures correspond to images x4.3 magnification (scale stands for 500μm) and lower pictures correspond to images with higher magnification, x28 (scale stands for 50μm). L, lumen of the glands.

In prostates from the control diet group, acini exhibited simple and columnar epithelium with papillary and tufting areas, and this was associated with low-grade PINs (Fig 3A and S3 Fig). Glands displayed marked ductal dilatation associated at some points with secretion-filled, more distended ducts as compared to WT prostates. As recently reported, DP and LP were more affected than VP [28]. No obvious histopathological feature discriminated prostate tumors of Pb-Prl mice fed with either milk type compared to water-fed control animals (Fig 3A and S3 Fig).

#### KIMAP mice

According to the expression pattern of Tag oncogene in KIMAP prostates (S2 Fig), DP and LP epithelium exhibited more pronounced and homogenously distributed alterations than VP. These included papillary and tufting pattern leading to low/medium-grade PIN lesions (Fig 3B and S4 Fig); these regions reflect local (in situ) neoplastic events, though without global disorganization of gland architecture. Similar histological description applied to the epithelium of KIMAP prostates from both milk groups, without any sign of aggravation (Fig 3B and S4 Fig).

Taken together, these results suggest that, in the conditions tested in these experiments, neither whole nor skim milk consumption noticeably affected the prostate histopathological phenotypes in these two models of genetically-induced prostate tumors. To challenge this initial observation, we assessed other quantifiable hallmarks of prostate tumor progression, including epithelial proliferation, inflammation, microinvasion and tumor markers specific to each model.

### High milk consumption decreases prostate cell proliferation

#### Pb-Prl mice

**Fig 4:**
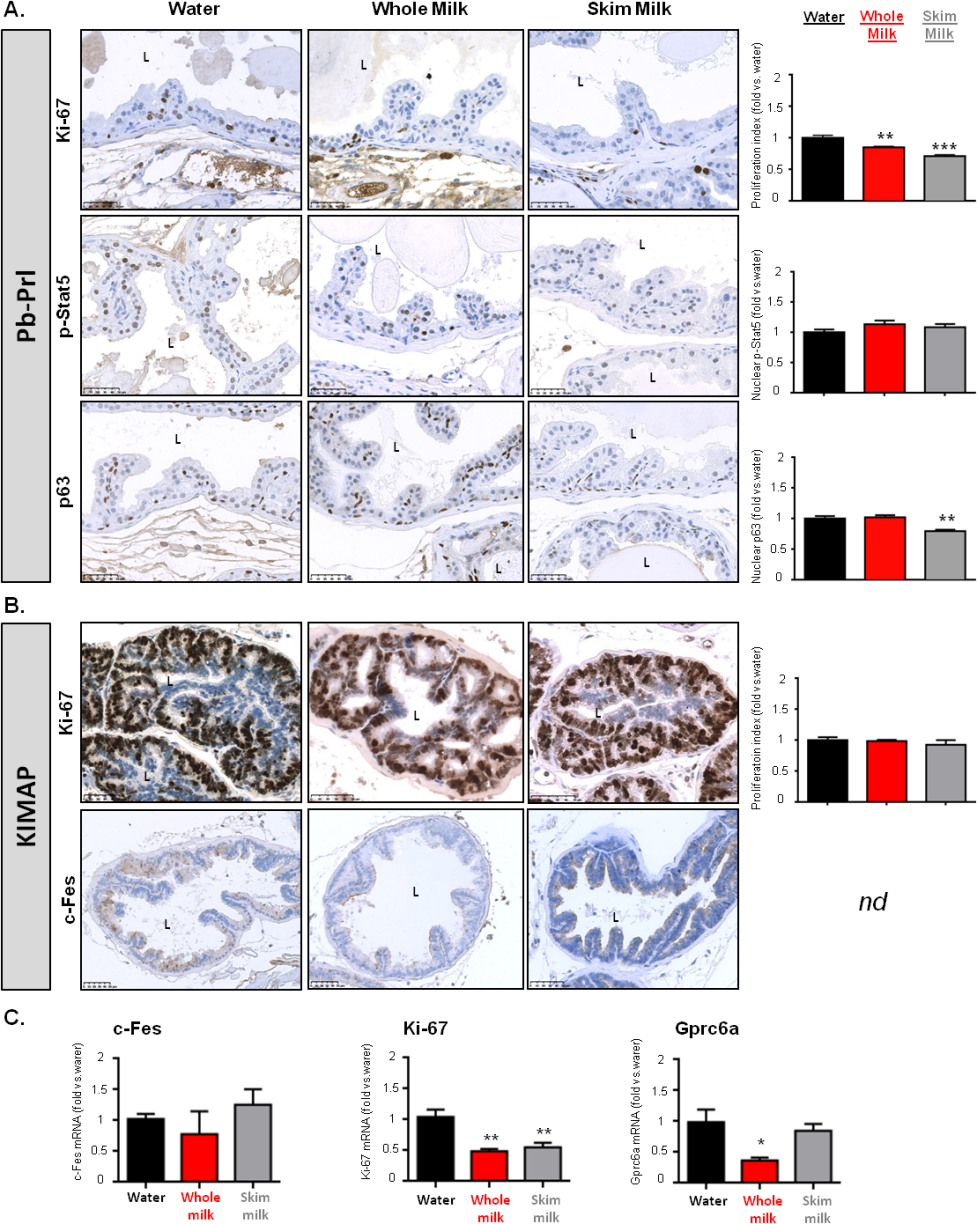
Effect of milk diets on expression of tumor markers. Various tumor markers relevant to Pb-Prl (A) and KIMAP (B and C) models were assessed in DP following 27- or 15-week milk diets respectively, as compared to control (water) group. Proliferation index (Ki-67 staining, shown for both models), activated Stat5 (p-Stat5) and p63 expression (for Pb-Prl mice) and c-Fes expression (for KIMAP mice) were assessed from immunohistochemical (IHC) analysis (n = 6 per group; pictures show representative staining, scale stands for 50μm). For each marker (except c-Fes), IHC were quantified and the results are represented on adjacent bar histograms and are expressed as fold change *vs*. control (water) group. L, lumen of the glands. (C) q RT-PCR analysis of c-Fes, Ki-67 and Gprc6a mRNA expression in DP of KIMAP mice following 15-week milk diets. Results are expressed as fold change *vs*. control (water) group and are shown as means ± S.D. P values are represented *vs*. water control group (*). *nd*, for not determined.

The proliferation index (PI), i.e. the ratio of Ki-67-positive *vs*. total epithelial cells, is often correlated to the clinical course of cancer. Accordingly, the PI of prostates harvested from Pb-Prl mice fed control diet was higher compared to WT prostates (18.8 ± 0.8% versus 2.5 ± 0.9%, respectively), but nevertheless much lower than in the rapidly progressing KIMAP prostate tumors (see below). Both milk types significantly reduced the global epithelial PI of Pb-Prl tumors (Fig 4A, upper panels), and this protective effect was very marked in the two most proliferative lobes, i.e. DP and VP (Table 4 and S5 Fig).

**Table 4:** Proliferation Index of the various prostate lobes in the two mouse models used in this study and in WT mice.

Basal PI (%, mean ± SD)	AP	DP	LP	VP
**Pb-Prl** (n = 6)	**13.54** ± 3.31	**23.35** ± 3.34	**9.15** ± 2.11	**21.20** ± 3.23
**KIMAP** (n = 6)	n.d.	**55.91** ± 4.70	**56.25** ± 4.27	**27.36** ± 4.74
**WT** (n = 3)	n.d.	**3.76** ± 0.21	**1.72** ± 0.26	**1.98** ± 0.33

PI: proliferation index; AP, anterior prostate; DP, dorsal prostate; LP, lateral prostate; VP, ventral prostate; n.d, not determined

#### KIMAP mice

KIMAP prostates from the control diet group display a very high epithelial PI (50 ± 2.9%), with DP and LP as the most proliferative lobes (Table 4); again, this correlated with the expression pattern of the SV40-Tag oncogene (S2 Fig). Similar Ki-67 staining patterns were observed in whole and skim milk groups as compared to water-fed control mice (Fig 4B, upper panels). Quantification of the epithelial PI with respect to the whole prostate (Fig 4B) or each individual lobe (S5 Fig) confirmed that high milk consumption had no effect on this parameter. Interestingly, when cell proliferation was assessed by measuring Ki-67 mRNA expression (which, in contrast to IHC, does not allow discriminating proliferation of the epithelium from that of other cell compartments), both milk types significantly reduced Ki-67 expression by ~50% in all lobes (Figs 4C and S4).

This first set of results suggests that high milk consumption globally reduces cell proliferation in prostate tumors of these two mouse models, which more specifically applies to the epithelial compartment in the Pb-Prl model.

### High milk consumption does not promote increased expression of tumor markers

We next investigated the expression of other tumor markers specifically relevant to each model. These included activated (phosphorylated) Stat5 and p63 expression for the Pb-Prl model, and c-Fes and Gprc6a expression for the KIMAP model.

#### Pb-Prl mice

We recently showed that Stat5 was the major signaling mediator of rPRL effects in Pb-Prl prostates [24,28]. Accordingly, the pattern of phospho-Stat5 exhibits a lobe-related intensity gradient (DP>LP>VP; AP not investigated in former studies) that nicely correlates the lobe-specific histological hallmarks of tumor development described above. Thus, we quantified the effects of milk regimens on Stat5 phosphorylation in all lobes, except VP that remained at background levels in all groups. Clearly, high milk consumption (whole or skim) did not impact on the global and lobe-specific levels of Stat5 activation (Figs 4A middle panels, and S4).

The p63 transcription factor is a member of the p53 family. In the prostate epithelium, p63 expression specifically identifies the basal cell compartment, which has been reported to host the prostate (cancer) stem cells in niche-like sites both in humans and mice [29,30]. We have recently shown that this basal/stem cell compartment was dramatically expanded in Pb-Prl prostate tumors [24], a phenotype tightly linked to Stat5 activation [28]. We then investigated whether tumor progression could be aggravated following milk diets through expansion of the basal/stem cell compartment. In all lobes we could not detect major staining differences in terms of p63 expression among the various diet groups, although staining appeared slightly reduced upon skim milk diet in the DP as shown in Fig 4A (lower panels). This was confirmed by staining quantification of p63 as shown in corresponding histograms (Figs 4A, bottom panels, and S4), with significantly reduced expression of p63 upon skim milk diet.

#### KIMAP mice

The proto–oncogene c-Fes is a non-receptor protein tyrosine kinase that regulates cell proliferation and inflammation; it was recently established as a new biomarker of Pca progression both in KIMAP mice and in human Pca [31]. Immunohistochemical analyses revealed basal levels of cytoplasmic c-Fes staining in the epithelium of KIMAP prostates (Fig 4B, bottom panels, and S5 Fig). Staining reproducibly appeared to be slightly stronger in skim milk diet group and slightly more discrete in whole milk group compared to water-fed animals, although these differences were minor (Figs 4B and S4). Analysis of c-Fes mRNA levels by q RT-PCR showed globally unaltered expression in milk regimen groups (Fig 4C).

Gprc6a is a novel molecular target for regulating prostate growth and cancer progression. Increments in Gprc6a may augment the ability of prostate cancer cells to proliferate in response to dietary- and bone-derived ligands [32]. As shown in Figs 4C and S4, Gprc6a mRNA expression was unaltered in skim milk group and even decreased in whole milk group compared to water-control group.

Taken together, these results clearly show that milk consumption does not promote expression of acknowledged markers of tumor progression, and can even have, in some cases, a protective effect.

### High milk consumption does not promote inflammation, fibrosis or tumor invasiveness

#### Inflammation

**Fig 5:**
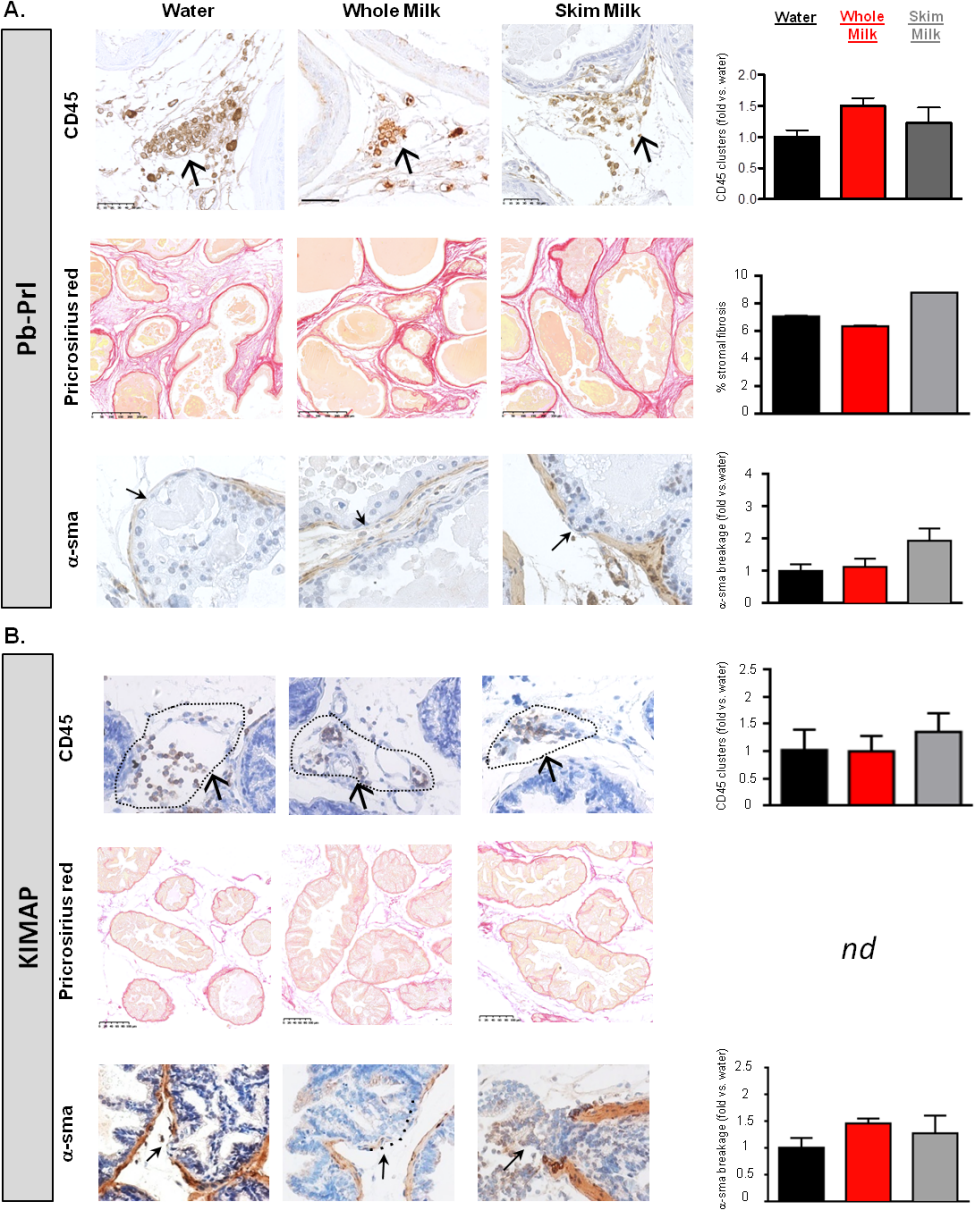
Inflammatory and tumor invasiveness status of prostates following milk diets. Inflammation and invasion were assessed in DP from Pb-Prl (A) and KIMAP (B) animals were following 27- or 15-week milk diets respectively, as compared to control (water) group. Immunohistochemical analysis of leukocytes-associated inflammation is represented in upper panels. Whole prostate sections of animals after milk diets were stained with anti-CD45 antibody and counterstained with haematoxylin. Images were taken at x39 magnification (scale stands for 50μm). One representative CD45 stained cells cluster is shown per picture as indicated by arrows. Inflammatory-associated fibrosis was assessed by histological pricrosirius red staining of sections from dorsal prostate lobe (middle panels). Images were taken at x10 or x16 magnification for Pb-Prl and KIMAP respectively (scales stand for 250 or 100μm) and areas of intense staining were measured. Microinvasion (lower panels) was evaluated by anti α-sma immunostaining from dorsal prostate sections and counterstained with haematoxylin. Images were taken at x40 magnification and ruptures in α-sma staining (as shown by arrows) were counted. All quantifications were done from n = 6 animals per group and are represented on bar histograms to adjacent pictures. Results are expressed as fold change *vs*. control (water) group and are shown as means ± S.D. *nd*, for not determined.

In prostate cancer, a growing body of evidence suggests a link between chronic or persistent inflammation and tumor development [33]. In particular, inflammation was suggested to be a key player in early events of tumor development. We thus addressed the question whether mice receiving milk diets could display differences in their inflammatory phenotype. We performed CD45 IHC staining in all groups and we quantified the number of CD45-positive clusters as a hallmark of the prostate inflammatory status (see upper panels of Fig 5A and 5B for Pb-Prl and KIMAP prostates, respectively). In KIMAP mice, inflammatory cells were quite rare and usually not more than 2 clusters were counted per half prostate, indicating the low-grade inflammatory status in this model. In contrast, in the Pb-Prl model, a moderate basal stromal inflammation has been reported by us and others, with a significant increased cluster number reported on average per half prostate [21,34]. In both models, the number of CD45-positive clusters was unchanged by both milk diets, suggesting that inflammation is neither induced (KIMAP) nor worsened (Pb-Prl) by high milk consumption.

#### Fibrosis

We recently observed that Pb-Prl prostates displayed stromal fibrosis as identified by pricrosirius red staining [34]. We here quantified this phenotype by image analysis as described in Methods, and found that fibrotic areas accounted for ~7% of total stroma area in Pb-Prl prostates; in contrast, KIMAP prostates did not display such a fibrotic phenotype (<1%, i.e. background level)(see middle panels of Fig 5A and 5B, respectively). None of the milk regimens affected these phenotypes.

#### Microinvasion

It is well recognized that the distinction between PIN and adenocarcinoma is whether the neoplastic cells penetrate through the basement membrane and invade into the surrounding stroma. Thus, degradation of the basement membrane and invasion of the stroma are critical steps in the tumor progression and the PIN-to-adenocarcinoma transition. Normal mouse prostate displays a thin rim of fibromuscular stroma surrounding individual glands. We can specifically identify this layer by staining with α-smooth muscle actin (α-sma) antibody. Thus, alteration of the basement membrane and subsequent invasion potency was assessed by the identification of the basement membrane breakages within the α-sma positive staining (producing a discontinuous staining pattern). Quantification of invasion was performed by counting the number of α-sma breakages. Results are shown in lower panels of Fig 5A and 5B for Pb-Prl and KIMAP prostates, respectively, and in S6 Fig for individual lobes. Expectedly, a very low number (<2) of α-sma breakages could be detected in Pb-Prl prostates, whereas more (up to 10) were counted in KIMAP prostates, in accordance with the neoplasic phenotype reported for this model. Following high milk diet consumption, no significant differences in the number of α-sma breakages could be measured in whole and skim milk groups as compared to control diet in both models. Overall, these results indicate that high milk diets do not confer more aggressive properties to intraepithelial (pre-) neoplastic lesions.

In conclusion, similarly to what was observed above for histology, proliferation index and tumor markers, all the other prostate phenotypes that we investigated, including inflammation, fibrosis and microinvasion, were unaffected by high milk consumption (Fig 5).

## Discussion

Although the impact of regular milk consumption on the risk of developing a Pca has been addressed in many observational studies, the conclusions are still a matter of intense debate among epidemiologists [5,6]. Strikingly, the consequences of milk consumption on the progression of diagnosed prostate tumors are much less documented (see below). This question appears particularly relevant with respect to benign tumors and early stages of Pca as these pathological states are typically considered as chronic diseases. The prevalence of BPH starts to rise from the age of 40 to achieve 50% of men at age 51–60 and up to 80% at the age of 85. It is marked by a strong increase in epithelial and/or stromal cell proliferation that can lead to nearly doubling volume of the transition zone by the age of 55–60 ([35] and references therein). With respect to Pca, prevalence also increases with age and starts to rise at age 40–50, but most of cancers detected are considered non-significant (of low stage and grade) and managed with active surveillance to avoid over-treatment [36]. Thus, as such patients may live decades with their non/poorly aggressive tumors, determining the environmental parameters that may favor or in contrast prevent tumor progression is obviously of clinical relevance.

To assess this issue experimentally, we undertook a large *in vivo* interventional study involving two genetically-modified mouse models representing early-stage benign (Pb-Prl) and (pre-) malignant (KIMAP) prostate tumorigenesis. Both models were well-suited to assess any dietary effects on tumor progression since, at their respective age of sacrifice, prostate tumors were still well-differentiated and displayed mainly low-grade (Pb-Prl) or low-to-medium grade (KIMAP) PINs [21,23]. Pb-Prl and KIMAP models were in fact very complementary for that purpose. First, although both exhibit proliferative characteristics, their progression rate is different (KIMAP > Pb-Prl). Second, they display different hallmarks of prostate tumor progression offering a large panel of relevant features to follow up in order to assess any effect of nutritional diets. On the one hand, Pb-Prl prostates exhibit stromal inflammation and activation of Stat5 pathway [21,24,28], both of which have been linked to prostate tumorigenesis (for reviews see Ref. [33,37]); of note, the moderate degree of inflammation in Pb-Prl tumors is particularly appropriate to identify anti- or pro-inflammatory signals [34]. On the other hand, KIMAP tumors exhibit frequent microinvasion features [23], and expression of the proto-oncogenes c-Fes and Gprc6a can be used as markers of Pca progression in this model [31,32]. Importantly, it was necessary to assess that milk regimens did not intrinsically interfere with the tumor drivers of each model in order to prevent biased interpretation when comparing the various diet groups. Indeed, the lobe-specific pattern and absolute levels of expression of Tag transgene (as well as of PSP94 gene in which Tag is inserted)[23] in KIMAP prostates was unaltered by milk diets. Likewise, the lobe-specific pattern and absolute levels of activated Stat5 in Pb-Prl tumors was also unaffected by the diets; in addition to account for the absence of tumor progression, this finding also reflected unaltered expression/activity of the rPrl transgene (S5 Fig). This is to our knowledge the first large-scale interventional study performed in mice that combines two types of milk diets and two models of genetically-induced prostate tumorigenesis.

Our results revealed that high consumption of milk (either whole or skim) for 15 to 27 weeks did not promote the progression of Pb-Prl and KIMAP prostate tumors as regards to the parameters tested. Indeed, when tumor-promoting effects were noticed on any of the parameters investigated, they were faint, model- and/or lobe-specific, which argued against their global relevance regarding tumor progression. As a typical example, expression of c-Fes oncogene in KIMAP tumors was slightly enhanced in LP upon skim milk diet (S5 Fig, qRT-PCR data), but this effect was not confirmed at the protein level (see IHC panels) neither observed in other lobes nor found under whole milk diet. As a hallmark of the absence of significant alteration due to milk consumption, the lobe-specific phenotypes of KIMAP tumors (due to differential Tag expression) were maintained in the various diet groups, thus strengthening the absence of inter-group differences. Unexpectedly, we observed that both milk types significantly reduced epithelial cell proliferation of Pb-Prl tumors (Fig 4). Since this was not observed in KIMAP mice, this suggests that milk consumption may exert a mild protective effect on moderately but not highly proliferating prostate tumors (see Table 4). The slightly reduced amplification of the basal/stem cell compartment under skim milk diet, as reflected by the lower p63 expression index, was also suggestive of a mild protective effect of this specific regimen. The overall effects of the milk diets in both transgenic models are summarized in S1 Table. These conclusions cannot be confronted to similar *in vivo* studies since this issue has not been experimentally tested by others. At best, a recent *in vitro* study reporting that caseins (typical milk proteins) could promote proliferation of PC3 and LNCaP prostate cancer cells argues against our results. However, these effects i) were obtained at extremely high casein concentration (1 mg/mL), ii) were not validated *in vivo* [38], and iii) only involved a single milk protein family out of the complex nature of ingredients constituting milk. Taken together, it is fair to claim that, globally, milk does not promote prostate tumor progression in Pb-Prl and KIMAP mice. Since the results obtained in both mouse models were very consistent, we believe that the absence of a strong milk effect is not specific to these models or to the tumor drivers involved, but more probably reflects the intrinsic properties of the diets. These conclusions are further supported by our ongoing studies showing that, in both mouse models, prostate tumorigenesis could be markedly accelerated by other types of diets given for the same duration (Bernichtein et al, manuscript in preparation).

Nevertheless, some limitations of our study should be considered to avoid over-interpretation of the results. First, we cannot exclude that longer duration of milk regimen and/or consumption of higher doses of milk might ultimately exert detrimental effects. In this respect, it must be stressed that the milk dose used in this study was already extremely high as it corresponds to ~10mL/day/mouse, which is within the top range of the mean daily water intake reported for classical mouse strains [39]. This high dose was chosen to compensate the relatively short time of regimen administration (15–27 weeks) compared to animal lifespan (~2 years). Careful extrapolation of such high daily milk intake to the human context should correspond to the upper range of recommended daily water intake, i.e ~2 L/day for an active adult man. In fact, it is probably unusual that adults ingest such high amounts of milk every day. Accordingly, epidemiological studies that have addressed the effects of high milk consumption on Pca risk (e.g. the EPIC study) involved milk intakes ranging from 2 to 3 cups/day (~500–750 mL/day). Thus, the absence of obvious strong adverse effects towards progression of benign or early-stage malignant prostate tumors in our study is unlikely to be due to insufficient doses of milk intake. Second, as reported in Table 1, the mean caloric intake was different for the three diets (Table 1A), as was also the density of milk-derived components in both milk types (Table 1B). Therefore, some interference is possible with the outcome of each regimen. For example, one cannot exclude that mild intrinsic inhibition of tumor growth by a given regimen may be masked by overall greater caloric intake, or inversely, that mild intrinsic enhancement of tumor growth may be balanced by overall caloric intake reduction. It is however very difficult to discriminate such opposite contributions as the caloric intake is intrinsically linked to the type of diet. Third, the relevance of our results to the human context should be considered with caution. Since mice never develop spontaneous prostate cancer, these results were obtained using genetically-modified mouse models, which are imperfect mimicry of human prostate tumorigenesis. Also, as prostate anatomy differs in many aspects between mice and humans, the mechanisms underlying the promoting or protective effects of diets on prostate tumor progression may display species-specificity.

As stated in the Introduction, the majority of human epidemiological studies that evaluated milk consumption as a risk factor for Pca focused on overall cancer *incidence* (see Table 62 of Ref. [6]), an issue that was not addressed in our study due to the full penetrance of prostate tumorigenesis in our transgenic models. A smaller number of human studies have evaluated milk consumption as a risk factor for Pca *mortality* [19,40–45]. Since mortality is an endpoint that encompasses cancer progression, factors associated with increased risk of mortality can be viewed as factors potentially associated with Pca progression. Although some of these studies concluded that milk intake (especially whole milk) was associated with a small elevation in risk of progression to fatal disease [19,40–42], these studies were not included in (or were excluded from) the latest Systematic Literature Review of the WCRF International's Continuous Update Project [5,6]. In fact, the meta-analysis of the studies that matched the WCRF inclusion criteria [43–45] concluded to the absence of association between milk intake and fatal Pca, with no separate judgment for low-fat or whole milk [5,6]. These conclusions are in good agreement with the results reported in our study.

In conclusion, we here report a unique *in vivo* study assessing the outcome of high consumption of milk diets on the progression of early-stage prostate tumors in mice. Our results argue for an absence of adverse effect. The putative protective effects observed on some parameters (see S1 Table) should not be over-interpreted since they were not uniform among mouse models and/or milk types. Definitive confirmation of our results awaits these experiments to be repeated using other settings, including longer timeframe and other mouse models of prostate tumorigenesis. Finally, caution is obviously required in extrapolating the results obtained using rodent models to the human context.

## Supporting Information

S1 Fig Weekly body weights of Pb-Prl (panel A) and KIMAP (panel B) mice in the three diet groups. Mouse weights in the three diet groups were measured once a week from 3 weeks of age (i.e. when regimens started) until sacrifice (30 weeks of age for Pb-Prl mice, 18 weeks of age for KIMAP mice).
(TIF)

S2 Fig Milk diets do not interfere with transgene expression in the KIMAP mouse model. SV40-Tag mRNA expression was assessed by q RT-PCR (A) and immunohistochemistry (B) in dorsal (DP), lateral (LP) and ventral (VP) lobes from KIMAP animals (n = 6/condition) following milk diets. PSP94 mRNA expression was analyzed by q RT-PCR (C) in the three prostate lobes. All results are expressed as fold expression *vs*. expression in control water group and are expressed as means ± S.D.
(TIF)

S3 Fig Milk diets do not affect prostate tumor histopathology in Pb-PRL mice. Histological analysis of lateral (LP), ventral (VP), and anterior (AP) prostates from Pb-PRL mice after a 27-week milk diet, as compared to control (water) group. For each panel, images are representative of the group (n = 10 mice per group). Sections were stained with haematoxylin/eosin (HE) and for each diet condition, pictures correspond to images x30 magnification (LP and VP), and x10 (AP). L, lumen of the glands. S, secretion. Arrowheads point to cribriform structures, and stars to pseudostratified epithelium.
(TIF)

S4 Fig Milk diets do not affect prostate tumor histopathology in KIMAP mice. Histological analysis of lateral (LP), ventral (VP) and prostates from KIMAP mice after a 15-week milk diets, as compared to control (water) group. For each panel, images are representative of the group (n = 10 mice per group). Sections were stained with haematoxylin/eosin (HE) and for each condition, pictures correspond to images x30 magnification. L, lumen of the glands.
(TIF)

S5 Fig Milk diets do not affect expression of prostate tumor markers. (A) Quantification of Ki-67 epithelial staining (proliferation index) in transgenic animals from each model and in WT C57BL/6J mice, as performed in all lobes of the prostate as indicated. Results are expressed in %, mean ± SD. *nd*, not determined. (B) IHC quantification of proliferation index, activated Stat5 and p63 expression in individual prostate lobes from Pb-Prl mice as indicated (n = 6). (C) Quantification of proliferation and c-Fes (by IHC and q RT-PCR) and Gprc6a in individual prostate lobes from KIMAP mice. For all quantifications, results are expressed as fold expression *vs*. water-CTL group.
(TIF)

S6 Fig Milk diets do not affect tumor invasiveness in any prostate lobe. Quantification of the number of α-sma breakages in Pb-Prl (A) and KIMAP (B) prostate lobes of mice on milk diets. Results are expressed as fold expression *vs*. water-CTL group as mean ± SD. AP, anterior prostate; DP, dorsal prostate; LP, lateral prostate and VP, ventral prostate.
(TIF)

S1 Table Summary of the protective and harmful effects of the milk diets in the Pb-Prl and KIMAP mouse models. (TIF)
