# Supplementary figures and images for: High Milk Consumption Does Not Affect Prostate Tumor Progression in Two Mouse Models of Benign and Neoplastic Lesions
Source: PLoS One. 2015 May 4;10(5):e0125423. doi: 10.1371/journal.pone.0125423 (PMC4418739; doi:10.1371/journal.pone.0125423)

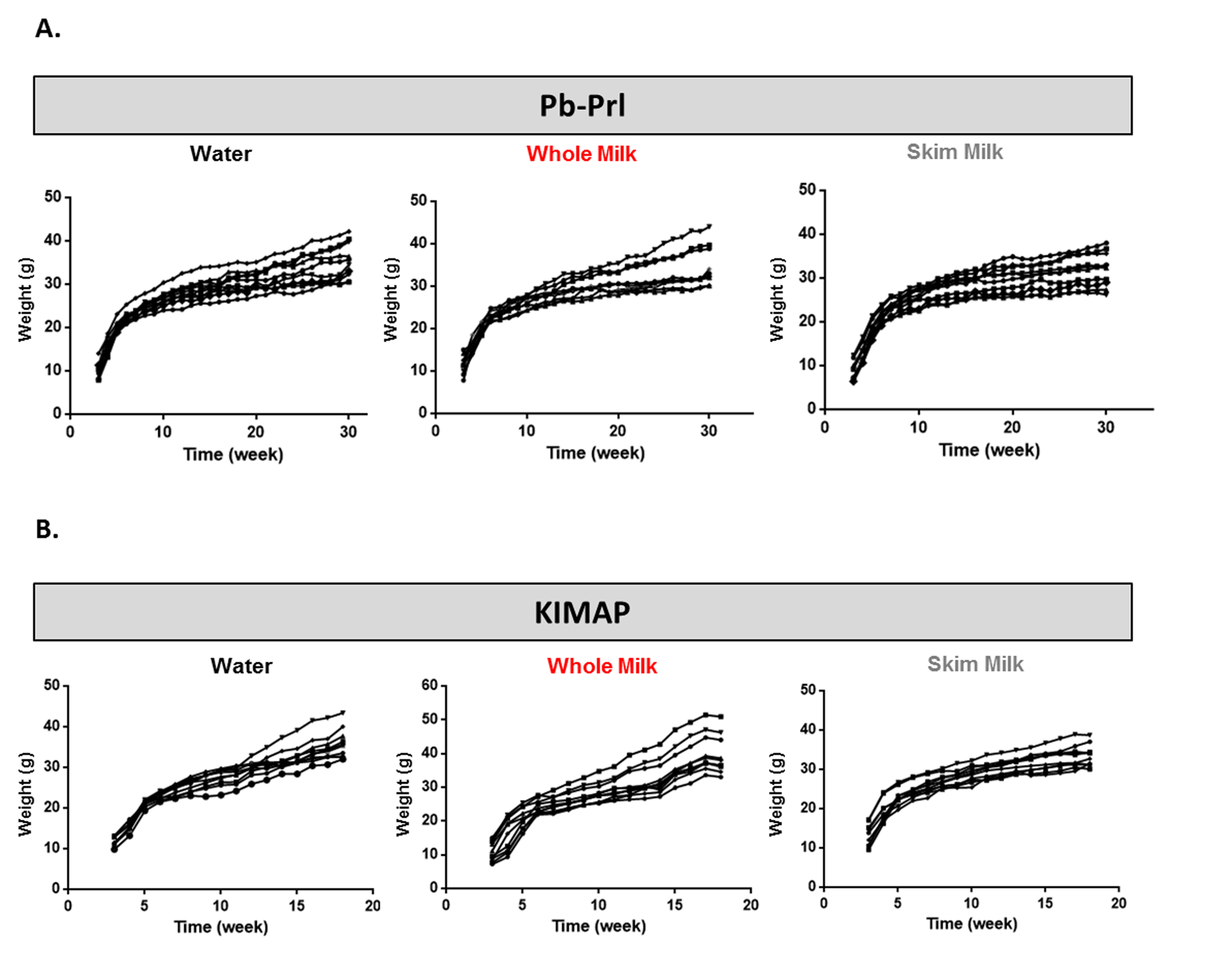

Supplement: S1 Fig — Mouse weights in the three diet groups were measured once a week from 3 weeks of age (i.e. when regimens started) until sacrifice (30 weeks of age for Pb-Prl mice, 18 weeks of age for KIMAP mice). (TIF) [file pone.0125423.s001.tif]

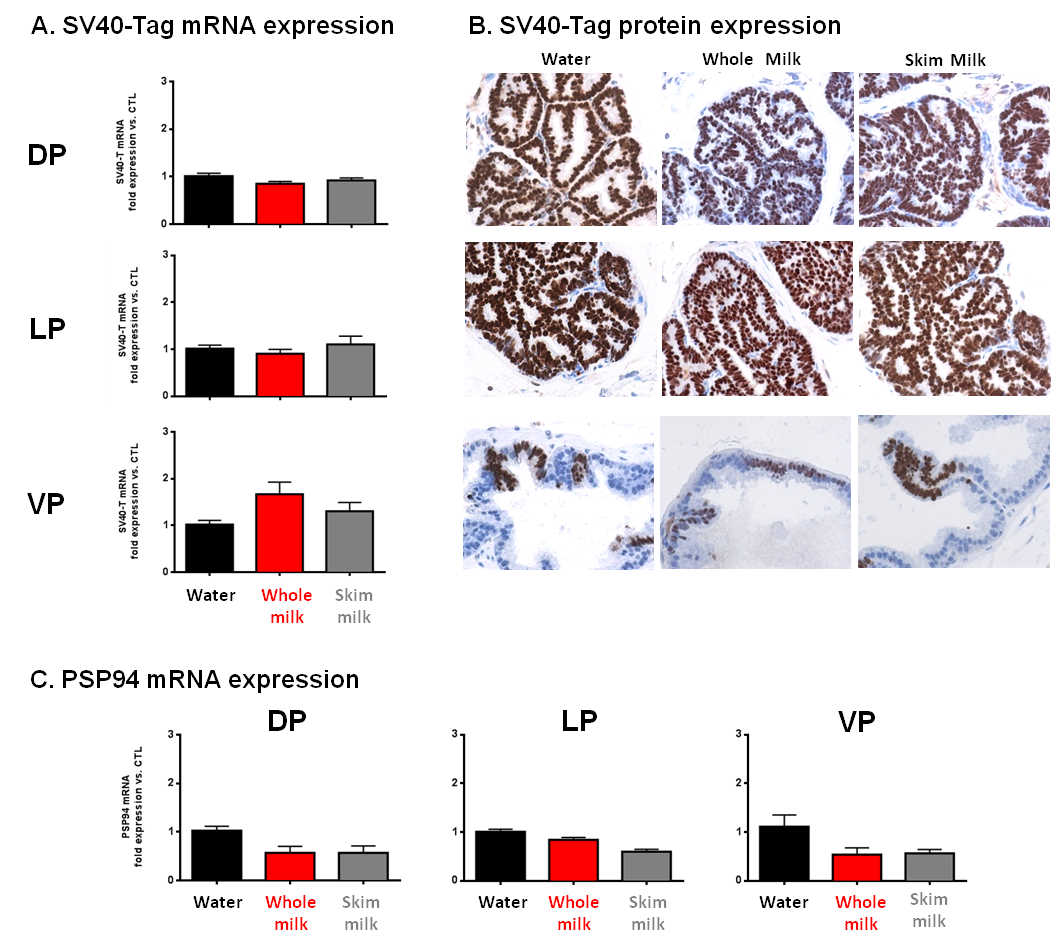

Supplement: S2 Fig — SV40-Tag mRNA expression was assessed by q RT-PCR (A) and immunohistochemistry (B) in dorsal (DP), lateral (LP) and ventral (VP) lobes from KIMAP animals (n = 6/condition) following milk diets. PSP94 mRNA expression was analyzed by q RT-PCR (C) in the three prostate lobes. All results are expressed as fold expression vs. expression in control water group and are expressed as means ± S.D. (TIF) [file pone.0125423.s002.tif]

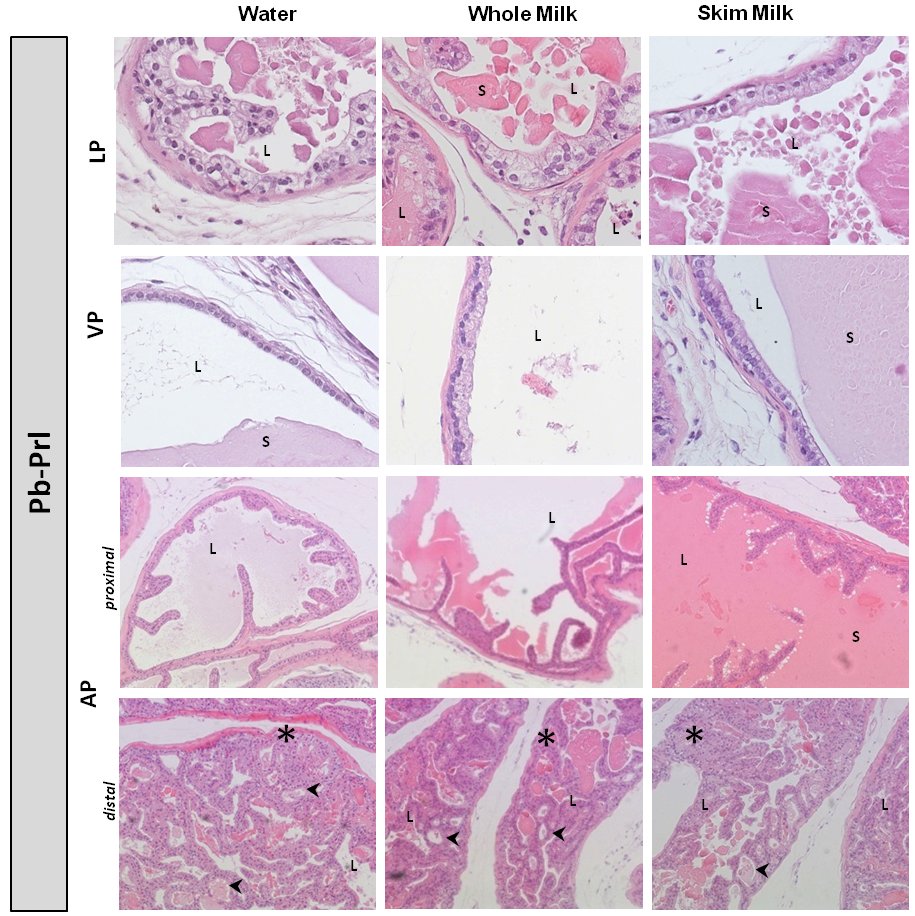

Supplement: S3 Fig — Histological analysis of lateral (LP), ventral (VP), and anterior (AP) prostates from Pb-PRL mice after a 27-week milk diet, as compared to control (water) group. For each panel, images are representative of the group (n = 10 mice per group). Sections were stained with haematoxylin/eosin (HE) and for each diet condition, pictures correspond to images x30 magnification (LP and VP), and x10 (AP). L, lumen of the glands. S, secretion. Arrowheads point to cribriform structures, and stars to pseudostratified epithelium. (TIF) [file pone.0125423.s003.tif]

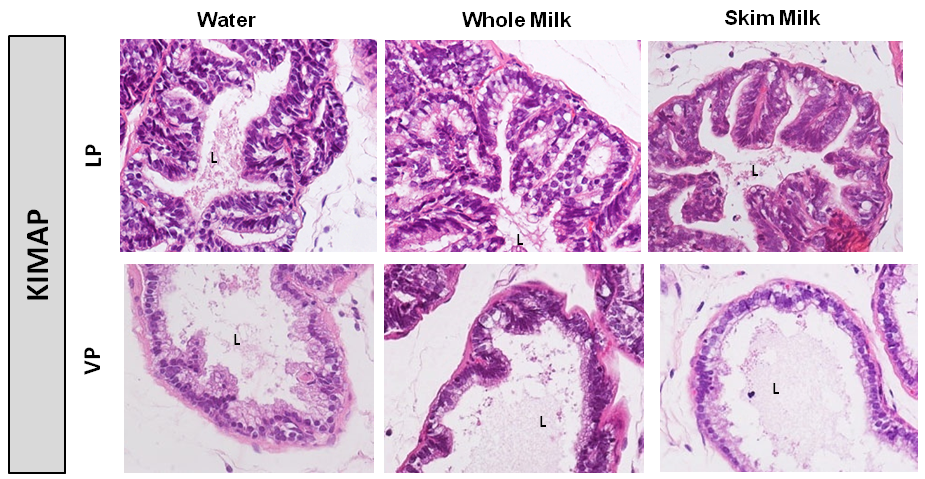

Supplement: S4 Fig — Histological analysis of lateral (LP), ventral (VP) and prostates from KIMAP mice after a 15-week milk diets, as compared to control (water) group. For each panel, images are representative of the group (n = 10 mice per group). Sections were stained with haematoxylin/eosin (HE) and for each condition, pictures correspond to images x30 magnification. L, lumen of the glands. (TIF) [file pone.0125423.s004.tif]

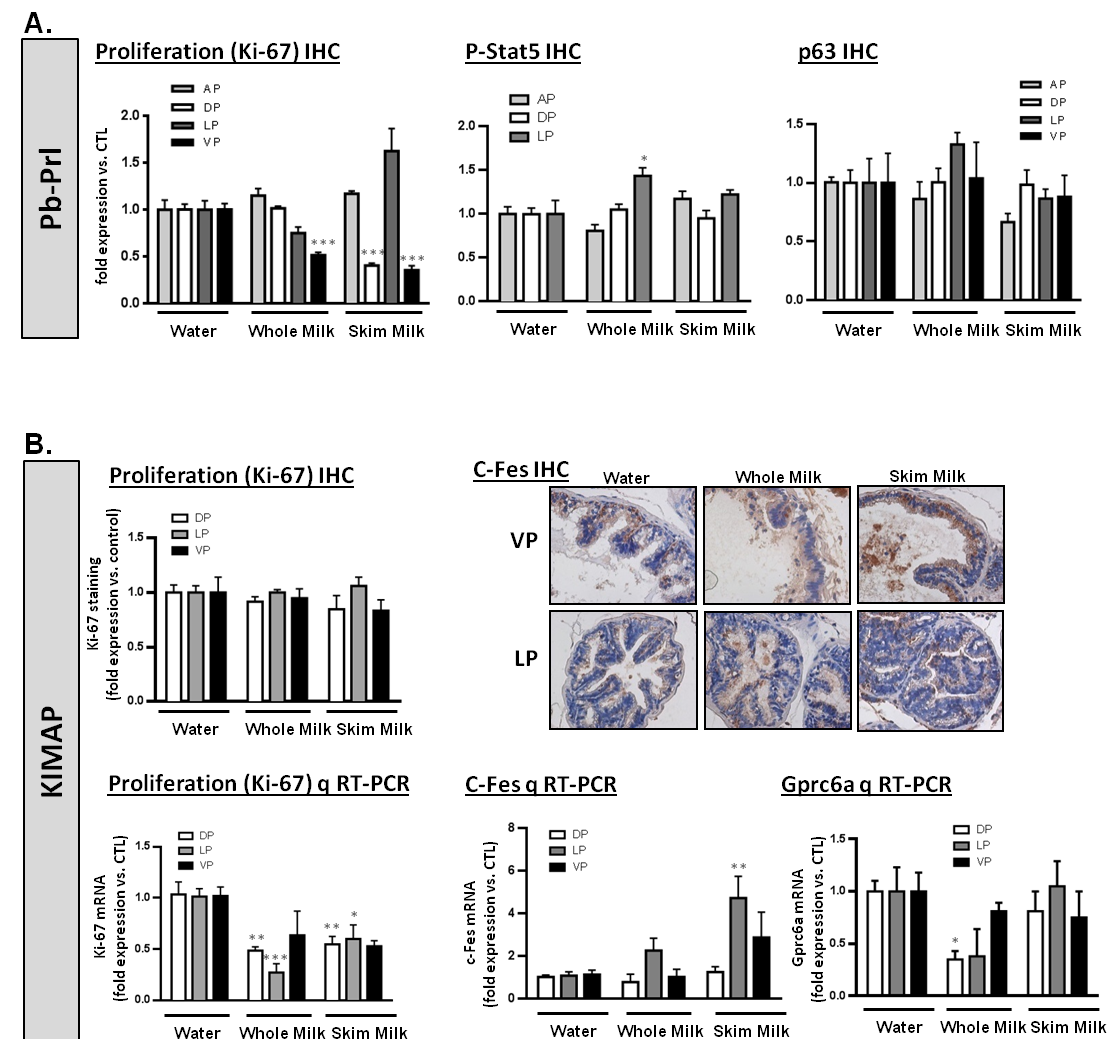

Supplement: S5 Fig — (A) Quantification of Ki-67 epithelial staining (proliferation index) in transgenic animals from each model and in WT C57BL/6J mice, as performed in all lobes of the prostate as indicated. Results are expressed in %, mean ± SD. nd, not determined. (B) IHC quantification of proliferation index, activated Stat5 and p63 expression in individual prostate lobes from Pb-Prl mice as indicated (n = 6). (C) Quantification of proliferation and c-Fes (by IHC and q RT-PCR) and Gprc6a in individual prostate lobes from KIMAP mice. For all quantifications, results are expressed as fold expression vs. water-CTL group. (TIF) [file pone.0125423.s005.tif]

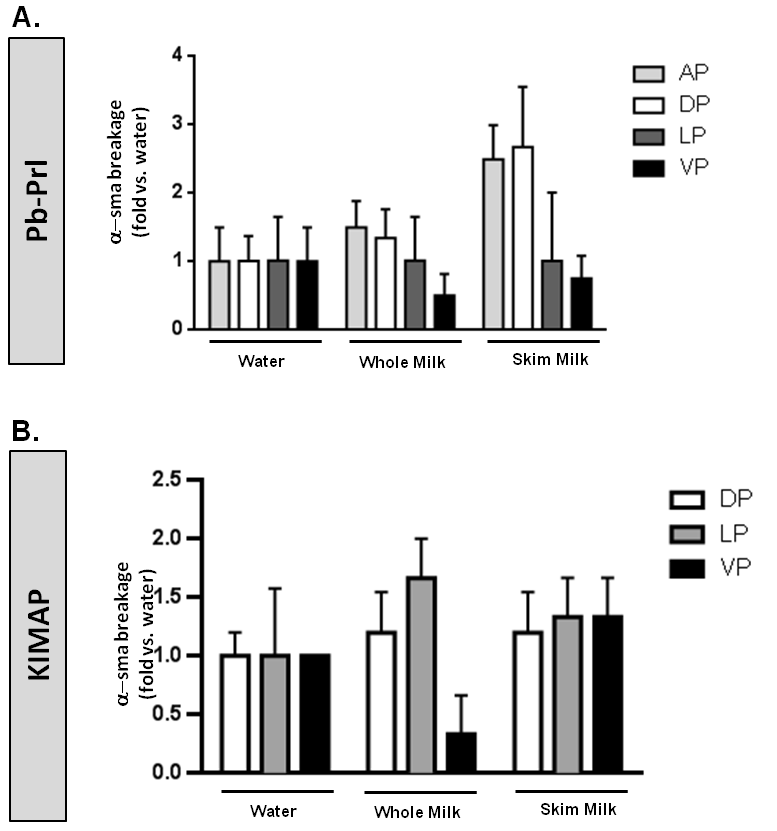

Supplement: S6 Fig — Quantification of the number of α-sma breakages in Pb-Prl (A) and KIMAP (B) prostate lobes of mice on milk diets. Results are expressed as fold expression vs. water-CTL group as mean ± SD. AP, anterior prostate; DP, dorsal prostate; LP, lateral prostate and VP, ventral prostate. (TIF) [file pone.0125423.s006.tif]

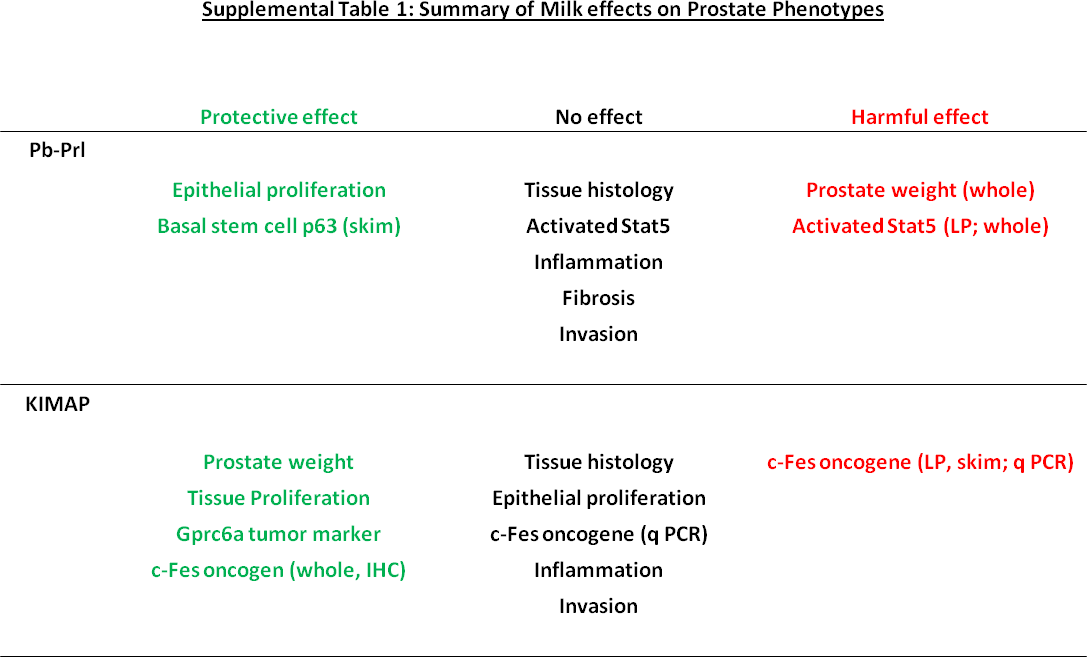

Supplement: S1 Table — (TIF) [file pone.0125423.s007.tif]
